# Supplementary material for: Executive functioning is associated to everyday interference of pain in patients with chronic pain
Source: PLoS One. 2024 Nov 15;19(11):e0313187. doi: 10.1371/journal.pone.0313187 (PMC11567537; doi:10.1371/journal.pone.0313187)
Supplement: S1 File — (DOCX) [file pone.0313187.s001.docx]

|  |  |  |  |  |  |  |
| --- | --- | --- | --- | --- | --- | --- |
| S1 Table: Multiple regression analysis for TAMPA Scale of Kinesiophobia | | | | | |  |
|  | B | Std. Error | Beta | t | Sig. | r2 = 0.12, p < 0.001 |
| (Constant) | 47.34 | 4.13 |  | 11.47 | **<0.001** |  |
| Age | -0.13 | 0.07 | -0.14 | -1.88 | 0.06 |  |
| Gender | 3.99 | 1.68 | 0.18 | 2.37 | **0.02** |  |
| Yearsofeducation | -0.63 | 0.26 | -0.18 | -2.4 | **0.02** |  |
| Summa depressivitetHAD initial | 0.47 | 0.15 | 0.23 | 3.19 | **0.00** |  |
| Siffrep_ poäng _bak | -0.21 | 0.3 | -0.05 | -0.71 | 0.48 |  |
|  |  |  |  |  |  |  |
| S2 Table: Multiple regression analysis for TAMPA Scale of Kinesiophobia | | | | | |  |
|  | B | Std. Error | Beta | t | Sig. | r2 = 0.15, p < 0.001 |
| (Constant) | 47.93 | 4.02 |  | 11.93 | **<0.001** |  |
| Age | -0.15 | 0.07 | -0.16 | -2.15 | **0.03** |  |
| Gender | 3.9 | 1.63 | 0.17 | 2.39 | **0.02** |  |
| Yearsofeducation | -0.55 | 0.25 | -0.16 | -2.19 | **0.03** |  |
| Summa depressivitetHAD initial | 0.48 | 0.14 | 0.24 | 3.34 | **0.00** |  |
| VerbalFluency | -0.07 | 0.05 | -0.11 | -1.44 | 0.15 |  |
|  |  |  |  |  |  |  |
| S3 Table: Multiple regression analysis for TAMPA Scale of Kinesiophobia | | | | | |  |
|  | B | Std. Error | Beta | t | Sig. | r2 = 0.15, p < 0.001 |
| (Constant) | 49.42 | 4.51 |  | 10.96 | **<0.001** |  |
| Age | -0.15 | 0.07 | -0.16 | -2.10 | **0.04** |  |
| Gender | 3.98 | 1.63 | 0.18 | 2.45 | **0.02** |  |
| Years of Education | -0.56 | 0.25 | -0.16 | -2.22 | **0.03** |  |
| HAD Depression | 0.44 | 0.15 | 0.22 | 2.95 | **0.00** |  |
| Semantic Fluency | -0.09 | 0.07 | -0.10 | -1.35 | 0.18 |  |
|  |  |  |  |  |  |  |
| S4 Table: Multiple regression analysis for TAMPA Scale of Kinesiophobia | | | | | |  |
|  | B | Std. Error | Beta | t | Sig. | r2 = 0.14, p < 0.001 |
| (Constant) | 49.03 | 4.59 |  | 10.68 | **<0.001** |  |
| Age | -0.15 | 0.07 | -0.16 | -2.22 | **0.03** |  |
| Gender | 3.80 | 1.65 | 0.17 | 2.31 | **0.02** |  |
| Years of Education | -0.56 | 0.26 | -0.16 | -2.18 | **0.03** |  |
| HAD Depression | 0.46 | 0.15 | 0.23 | 3.12 | **0.002** |  |
| Switching Fluency | -0.24 | 0.21 | -0.09 | -1.12 | 0.27 |  |
|  |  |  |  |  |  |  |
| S5 Table: Multiple regression analysis for TAMPA Scale of Kinesiophobia | | | | | |  |
|  | B | Std. Error | Beta | t | Sig. | r2 = 0.15, p < 0.001 |
| (Constant) | 41.32 | 4.49 |  | 9.2 | **<0.001** |  |
| Age | -0.15 | 0.07 | -0.16 | -2.15 | **0.03** |  |
| Gender | 4.11 | 1.65 | 0.18 | 2.50 | **0.01** |  |
| Years of Education | -0.56 | 0.25 | -0.16 | -2.20 | **0.03** |  |
| HAD Depression | 0.39 | 0.15 | 0.20 | 2.65 | **0.01** |  |
| CWIT - Inhibition | 0.09 | 0.04 | 0.17 | 2.25 | **0.03** |  |
|  |  |  |  |  |  |  |
| S6 Table: Multiple regression analysis for TAMPA Scale of Kinesiophobia | | | | | |  |
|  | B | Std. Error | Beta | t | Sig. | r2 = 0.17, p < 0.001 |
| (Constant) | 38.43 | 4.56 |  | 8.42 | **<0.001** |  |
| Age | -0.14 | 0.07 | -0.15 | -2.05 | **0.04** |  |
| Gender | 4.14 | 1.62 | 0.18 | 2.56 | **0.01** |  |
| Years of Education | -0.50 | 0.25 | -0.15 | -2.00 | **0.05** |  |
| HAD Depression | 0.42 | 0.14 | 0.21 | 2.94 | **0.004** |  |
| CWIT - Switching | 0.10 | 0.03 | 0.23 | 3.19 | **0.002** |  |
|  |  |  |  |  |  |  |
| S7 Table: Multiple regression analysis for MPI Interference | | | |  |  |  |
|  | B | Std. Error | Beta | t | Sig. | r2 = 0.18, p < 0.001 |
| (Constant) | 3.60 | 0.54 |  | 6.74 | **<0.001** |  |
| Age | 0.01 | 0.01 | 0.05 | 0.80 | 0.43 |  |
| Gender | 0.05 | 0.21 | 0.02 | 0.22 | 0.83 |  |
| Years of Education | -0.03 | 0.03 | -0.07 | -0.93 | 0.35 |  |
| HAD Depression | 0.11 | 0.02 | 0.42 | 6.14 | **<0.001** |  |
| Digit Span Backwards | -0.04 | 0.04 | -0.07 | -1.02 | 0.31 |  |
|  |  |  |  |  |  |  |
| S8 Table: Multiple regression analysis for MPI Interference | | | |  |  |  |
|  | B | Std. Error | Beta | t | Sig. | r2 = 0.20, p < 0.001 |
| (Constant) | 3.71 | 0.52 |  | 7.09 | **<0.001** |  |
| Age | 0.01 | 0.01 | 0.07 | 0.99 | 0.33 |  |
| Gender | -0.02 | 0.21 | -0.01 | -0.11 | 0.91 |  |
| Years of Education | -0.03 | 0.03 | -0.05 | -0.78 | 0.44 |  |
| HAD Depression | 0.11 | 0.02 | 0.42 | 6.17 | **<0.001** |  |
| Word Fluency | -0.01 | 0.01 | -0.14 | -2.02 | **0.05** |  |
|  |  |  |  |  |  |  |
| S9 Table: Multiple regression analysis for MPI Interference | | | |  |  |  |
|  | B | Std. Error | Beta | t | Sig. | r2 = 0.20, p < 0.001 |
| (Constant) | 4.07 | 0.58 |  | 7.08 | **<0.001** |  |
| Age | 0.01 | 0.01 | 0.08 | 1.11 | 0.27 |  |
| Gender | -0.01 | 0.20 | -0.00 | -0.05 | 0.96 |  |
| Years of Education | -0.03 | 0.03 | -0.05 | -0.74 | 0.46 |  |
| HAD Depression | 0.10 | 0.02 | 0.38 | 5.55 | **<0.001** |  |
| Semantic Fluency | -0.02 | 0.01 | -0.16 | -2.34 | **0.02** |  |
|  |  |  |  |  |  |  |
| S10 Table: Multiple regression analysis for MPI Interference | | | |  |  |  |
|  | B | Std. Error | Beta | t | Sig. | r2 = 0.20, p < 0.001 |
| (Constant) | 4.07 | 0.58 |  | 7.08 | **<0.001** |  |
| Age | 0.01 | 0.01 | 0.08 | 1.11 | 0.27 |  |
| Gender | -0.01 | 0.20 | -0.00 | -0.05 | 0.96 |  |
| Years of Education | -0.03 | 0.03 | -0.05 | -0.74 | 0.46 |  |
| HAD Depression | 0.10 | 0.02 | 0.38 | 5.55 | **<0.001** |  |
| Switching Fluency | -0.02 | 0.01 | -0.16 | -2.34 | **0.02** |  |
|  |  |  |  |  |  |  |
| S11 Table: Multiple regression analysis for MPI Interference | | | |  |  |  |
|  | B | Std. Error | Beta | t | Sig. | r2 = 0.18, p < 0.001 |
| (Constant) | 3.02 | 0.58 |  | 5.21 | **<0.001** |  |
| Age | 0.01 | 0.01 | 0.04 | 0.63 | 0.53 |  |
| Gender | 0.04 | 0.21 | 0.01 | 0.19 | 0.85 |  |
| Years of Education | -0.03 | 0.03 | -0.07 | -0.95 | 0.35 |  |
| HAD Depression | 0.11 | 0.02 | 0.39 | 5.70 | **<0.001** |  |
| CWIT - Inhibition | 0.01 | 0.01 | 0.10 | 1.38 | 0.17 |  |
|  |  |  |  |  |  |  |
| S12 Table: Multiple regression analysis for MPI Interference | | | |  |  |  |
|  | B | Std. Error | Beta | t | Sig. | r2 = 0.19, p < 0.001 |
| (Constant) | 2.95 | 0.60 |  | 4.96 | **<0.001** |  |
| Age | 0.01 | 0.01 | 0.05 | 0.75 | 0.46 |  |
| Gender | 0.04 | 0.21 | 0.01 | 0.19 | 0.85 |  |
| Years of Education | -0.03 | 0.03 | -0.06 | -0.91 | 0.37 |  |
| HAD Depression | 0.11 | 0.02 | 0.41 | 6.03 | **<0.001** |  |
| CWIT - Switching | 0.01 | 0.00 | 0.10 | 1.44 | 0.15 |  |
|  |  |  |  |  |  |  |

| S13 Table: Multiple regression analysis for MPI Life Control | | | |  |  |  |
| --- | --- | --- | --- | --- | --- | --- |
|  | B | Std. Error | Beta | t | Sig. | r2 = 0.23, p < 0.001 |
| (Constant) | 2.97 | 0.51 |  | 5.79 | **<0.001** |  |
| Age | 0.01 | 0.01 | 0.08 | 1.25 | 0.21 |  |
| Gender | 0.00 | 0.20 | 0.00 | 0.02 | 0.99 |  |
| Years of Education | 0.03 | 0.03 | 0.07 | 0.97 | 0.33 |  |
| HAD Depression | -0.13 | 0.02 | -0.47 | -7.14 | **<0.001** |  |
| Digit Span Backwards | -0.01 | 0.04 | -0.01 | -0.15 | 0.88 |  |
|  |  |  |  |  |  |  |
| S14 Table: Multiple regression analysis for MPI Life Control | | | |  |  |  |
|  | B | Std. Error | Beta | t | Sig. | r2 = 0.25, p < 0.001 |
| (Constant) | 2.75 | 0.50 |  | 5.50 | **<0.001** |  |
| Age | 0.01 | 0.01 | 0.08 | 1.16 | 0.25 |  |
| Gender | 0.02 | 0.20 | 0.01 | 0.08 | 0.93 |  |
| Years of Education | 0.02 | 0.03 | 0.04 | 0.61 | 0.55 |  |
| HAD Depression | -0.13 | 0.02 | -0.48 | -7.32 | **<0.001** |  |
| Word Fluency | 0.01 | 0.01 | 0.10 | 1.50 | 0.13 |  |
|  |  |  |  |  |  |  |
| S15 Table: Multiple regression analysis for MPI Life Control | | | |  |  |  |
|  | B | Std. Error | Beta | t | Sig. | r2 = 0.25, p < 0.001 |
| (Constant) | 2.41 | 0.55 |  | 4.40 | **<0.001** |  |
| Age | 0.01 | 0.01 | 0.07 | 1.03 | 0.30 |  |
| Gender | 0.01 | 0.20 | 0.00 | 0.05 | 0.96 |  |
| Years of Education | 0.02 | 0.03 | 0.04 | 0.53 | 0.60 |  |
| HAD Depression | -0.12 | 0.02 | -0.45 | -6.76 | **<0.001** |  |
| Semantic Fluency | 0.02 | 0.01 | 0.14 | 2.03 | **0.04** |  |
|  |  |  |  |  |  |  |
| S16 Table: Multiple regression analysis for MPI Life Control | | | |  |  |  |
|  | B | Std. Error | Beta | t | Sig. | r2 = 0.26, p < 0.001 |
| (Constant) | 2.26 | 0.56 |  | 4.08 | **<0.001** |  |
| Age | 0.01 | 0.01 | 0.08 | 1.14 | 0.26 |  |
| Gender | 0.06 | 0.20 | 0.02 | 0.31 | 0.76 |  |
| Years of Education | 0.01 | 0.03 | 0.02 | 0.33 | 0.74 |  |
| HAD Depression | -0.12 | 0.02 | -0.46 | -6.96 | **<0.001** |  |
| Switching Fluency | 0.06 | 0.03 | 0.17 | 2.46 | **0.02** |  |
|  |  |  |  |  |  |  |

| S17 Table: Multiple regression analysis for MPI Life Control | | | |  |  |  |
| --- | --- | --- | --- | --- | --- | --- |
|  | B | Std. Error | Beta | t | Sig. | r2 = 0.23, p < 0.001 |
| (Constant) | 3.12 | 0.56 |  | 5.6 | **<0.001** |  |
| Age | 0.01 | 0.01 | 0.09 | 1.32 | 0.19 |  |
| Gender | -0.01 | 0.20 | -0.01 | -0.07 | 0.95 |  |
| Years of Education | 0.03 | 0.03 | 0.06 | 0.84 | 0.40 |  |
| HAD Depression | -0.12 | 0.02 | -0.46 | -6.89 | **<0.001** |  |
| CWIT - Inhibition | -0.00 | 0.01 | -0.04 | -0.62 | 0.54 |  |
|  |  |  |  |  |  |  |
| S18 Table: Multiple regression analysis for MPI Life Control | | | |  |  |  |
|  | B | Std. Error | Beta | t | Sig. | r2 = 0.23, p < 0.001 |
| (Constant) | 3.23 | 0.57 |  | 5.64 | **<0.001** |  |
| Age | 0.01 | 0.01 | 0.09 | 1.29 | 0.20 |  |
| Gender | -0.02 | 0.20 | -0.01 | -0.09 | 0.93 |  |
| Years of Education | 0.03 | 0.03 | 0.05 | 0.77 | 0.44 |  |
| HAD Depression | -0.13 | 0.02 | -0.47 | -7.12 | **<0.001** |  |
| CWIT - Switching | -0.00 | 0.00 | -0.06 | -0.91 | 0.36 |  |
